# Dietary Effects on Gut Microbial Metabolism and Intestinal Inflammation in Mammals

**DOI:** 10.3390/metabo13091025

**Published:** 2023-09-20

**Authors:** Bei Gao, Haoyu Liu

**Affiliations:** 1 School of Marine Sciences, Nanjing University of Information Science and Technology, Nanjing 210044, China; bgao@nuist.edu.cn; 2 Key Laboratory of Hydrometeorological Disaster Mechanism and Warning of Ministry of Water Resources, Nanjing University of Information Science and Technology, Nanjing 210044, China; 3 College of Animal Science and Technology, Yangzhou University, Yangzhou 225009, China; 4 Joint International Research Laboratory of Agricultural & Agri-Product Safety, The Ministry of Education of China, Yangzhou University, Yangzhou 225009, China

Growing evidence has proven that the gut microbiota has a tremendous impact on mammalian health. The gut microbiota and microbial metabolism could be affected by many factors, among which dietary factors have drawn increasing interest from both the scientific community and the public. Exploring the dietary effects on gut microbial metabolism and intestinal inflammation is helpful for both disease prevention and overall well-being. This Special Issue of *Metabolites,* entitled “Dietary Effects on Gut Microbial Metabolism and Intestinal Inflammation in Mammals”, contains five original research articles, which cover the dietary effects of both natural bioactive compounds, including black raspberries, berry-derived cyanidin-3-glucoside, cysteine proteases from pineapple and papaya, and the combination of geranylgeraniol and green tea polyphenols, and an endogenous metabolite, lithocholic acid, which is a secondary bile acid derived from cholesterol.

Regarding the effect of black raspberries, Tu et al. observed that the consumption of black raspberries restored and modified the microbial changes induced by arsenic exposure (10 ppm in drinking water), including changes in the composition of the gut microbiota and its functional metabolites in C57BL/6 mouse model [1]. Anthocyanins are abundant in berries. In a follow-up study, the authors investigated the effects of a class of anthocyanins, cyanidin-3-glucoside, on the composition and functions of the gut microbiota in C57BL/6 mice [2]. Supplementation of cyanidin-3-glucoside through oral gavage induced changes in the composition of the gut microbiota and microbial metabolites. In particular, microbial metabolites which were involved in protein digestion and absorption were significantly altered by cyanidin-3-glucoside supplementation.

Bromelain and papain are cysteine proteases isolated from pineapple and papaya latex, which have various applications in medicine. Kostiuchenko et al. assessed the effect of bromelain and papain on the protein-digestive capacity, intestinal morphology and the composition of the gut microbiota in C57BL/6 mice [3]. The gut microbial composition was modulated by bromelain and papain supplementation. For instance, the level of beneficial *Akkermansia muciniphila* was increased, while the elevation of pancreatic trypsin activity was also observed in both bromelain-treated mice and papain treated mice. It is noteworthy that the thickness of the ileal mucosa was only increased by bromelain treatment. Furthermore, the authors used a human reconstructed 3D tissue model EpiIntestinal (SMI-100) to study the effects of 0.1, 1 and 10 mg/mL doses of each enzyme on tissue integrity and mucosal permeability. The highest doses of each enzyme increased paracellular permeability, while lower concentrations did not cause any significant change. Decreased tissue integrity was induced by bromelain at 60 min, while no significant difference was observed at other time points or lower concentrations. Tissue viability was not affected by any enzyme at the highest concentration after the exposure period of 1 h.

Shen et al. investigated the combinational effects of geranylgeraniol and green tea polyphenols on high-fat-diet-induced muscle atrophy and the composition of the gut microbiota in male C57BL/6J mice [4]. The consumption of a combination of geranylgeraniol and green tea polyphenols significantly reduced the body and fat mass, while increasing the normalized skeletal muscle mass. Elevated citrate synthase activity and reduced lipid peroxidation were observed in the soleus muscle of the mice fed with a combinational diet of geranylgeraniol and green tea polyphenols. Alpha diversity was not significantly altered by the supplementation, while beta diversity was significantly different between different groups. The relative abundance of *A. muciniphila* and *Subdoligranulum variabile* was increased, while the abundance of *Sporobacter termitidis* was decreased by the combinational supplementation.

Lastly, a study on the effect of an endogenous metabolite, lithocholic acid, was included in this Special Issue. Li et al. investigated the effects of a secondary bile acid, lithocholic acid, on deoxynivalenol-induced lethal cholesterol metabolic abnormality in intestinal epithelial cells (IPI-21) [5]. Deoxynivalenol is one of the most widespread mycotoxins. The authors found that lithocholic acid pretreatment alleviated the reduction in cell number induced by deoxynivalenol exposure. Mechanically, through the reduction in cleaved caspase 3 and cleaved PARP-1 expression, lithocholic acid restored the cell apoptosis induced by deoxynivalenol. Furthermore, lithocholic acid co-treatment significantly reduced the cellular level of cholesterol and bile acid contents as elevated by deoxynivalenol exposure. Transcriptomics analysis revealed that lithocholic acid inhibited the expression of key genes involved in the cholesterol biosynthesis and bile acid transformation in cells exposed to deoxynivalenol, suggesting that lithocholic acid ameliorated deoxynivalenol-induced apoptosis through maintaining the homeostasis of cholesterol metabolism in IPI-21 cells.

The present Special Issue of *Metabolites* summarizes the current progress on the dietary effects of natural bioactive compounds and endogenous metabolites on gut microbial metabolism and intestinal inflammation in mammals. These studies provide evidence for the development of dietary interventions or therapeutic approaches for disease prevention or treatment. More research is needed to explore the causal role that the gut microbiota plays during the dietary intervention with a sex-balanced study design to advance the scientific knowledge in this area.
